# Superior Verbal Memory Outcome After Stereotactic Laser Amygdalohippocampotomy

**DOI:** 10.3389/fneur.2021.779495

**Published:** 2021-12-09

**Authors:** Daniel L. Drane, Jon T. Willie, Nigel P. Pedersen, Deqiang Qiu, Natalie L. Voets, Scott R. Millis, Bruno P. Soares, Amit M. Saindane, Ranliang Hu, Michelle S. Kim, Kelsey C. Hewitt, Shahin Hakimian, Thomas Grabowski, Jeffrey G. Ojemann, David W. Loring, Kimford J. Meador, Edward Faught, John W. Miller, Robert E. Gross

**Affiliations:** ^1^Department of Neurology, Emory University School of Medicine, Atlanta, GA, United States; ^2^Department of Pediatrics, Emory University School of Medicine, Atlanta, GA, United States; ^3^Department of Neurology, University of Washington School of Medicine, Seattle, WA, United States; ^4^Department of Neurosurgery, Washington University School of Medicine, St. Louis, MO, United States; ^5^Coulter Department of Biomedical Engineering, Emory University, Atlanta, GA, United States; ^6^Department of Radiology and Imaging Sciences, Emory University School of Medicine, Atlanta, GA, United States; ^7^Nuffield Department of Clinical Neurosciences, Oxford Centre for Functional MRI of the Brain, University of Oxford, Oxford, United Kingdom; ^8^Department of Physical Medicine and Rehabilitation, Wayne State University School of Medicine, Detroit, MI, United States; ^9^Department of Radiology, University of Vermont Medical Center, Burlington, VT, United States; ^10^Department of Neurological Surgery, University of Washington School of Medicine, Seattle, WA, United States; ^11^Department of Neurology, Stanford University School of Medicine, Stanford, CA, United States; ^12^Department of Neurosurgery, Emory University School of Medicine, Atlanta, GA, United States

**Keywords:** laser interstitial thermal therapy (LITT), verbal memory outcome, open resection epilepsy surgery, hippocampal function, neural substrates of memory

## Abstract

**Objective:** To evaluate declarative memory outcomes in medically refractory epilepsy patients who underwent either a highly selective laser ablation of the amygdalohippocampal complex or a conventional open temporal lobe resection.

**Methods:** Post-operative change scores were examined for verbal memory outcome in epilepsy patients who underwent stereotactic laser amygdalohippocampotomy (SLAH: *n* = 40) or open resection procedures (*n* = 40) using both reliable change index (RCI) scores and a 1-SD change metric.

**Results:** Using RCI scores, patients undergoing open resection (12/40, 30.0%) were more likely to decline on verbal memory than those undergoing SLAH (2/40 [5.0%], *p* = 0.0064, Fisher's exact test). Patients with language dominant procedures were much more likely to experience a significant verbal memory decline following open resection (9/19 [47.4%]) compared to laser ablation (2/19 [10.5%], *p* = 0.0293, Fisher's exact test). 1 SD verbal memory decline frequently occurred in the open resection sample of language dominant temporal lobe patients with mesial temporal sclerosis (8/10 [80.0%]), although it rarely occurred in such patients after SLAH (2/14, 14.3%) (*p* = 0.0027, Fisher's exact test). Memory improvement occurred significantly more frequently following SLAH than after open resection.

**Interpretation:** These findings suggest that while verbal memory function can decline after laser ablation of the amygdalohippocampal complex, it is better preserved when compared to open temporal lobe resection. Our findings also highlight that the dominant hippocampus is not uniquely responsible for verbal memory. While this is at odds with our simple and common heuristic of the hippocampus in memory, it supports the findings of non-human primate studies showing that memory depends on broader medial and lateral TL regions.

## Introduction

We have demonstrated that minimally invasive surgery for temporal lobe epilepsy (TLE), selective MRI thermography-guided interstitial thermal ablation of the amygdala and hippocampus (stereotactic laser amygdalohippocampotomy, SLAH), results in better naming and object recognition outcomes compared to open temporal lobe (TL) resection (1). This better outcome likely results from reducing “collateral damage,” sparing lateral and anterior TL structures and white matter pathways presumed to support these cognitive functions when accessing the medial TL (2, 3). Initial publications examining declarative verbal memory outcomes, a function long associated with medial TL regions, particularly the hippocampus, have suggested that this function can decline after laser ablation of the hippocampus (4–6). However, these studies lacked a comparison open resection group, and when aggregating the number of subjects showing decline across these papers, the percentage of patients declining with SLAH is much less than historically reported outcomes with open resection (7). Moreover, these earlier memory publications on SLAH differ on several key factors, including the metric of change used in the studies (reliable change index scores vs. 1 SD change). The original work involving patient H.M. has long suggested that structures beyond the hippocampus contribute uniquely to memory (8, 9), and this view is strongly supported by findings from animal and experimental research (10–12). Non-human primates typically require damage to extra-amygdalohippocampal structures to suffer significant memory dysfunction. Therefore, this paper will examine effects of surgery on declarative verbal memory by directly comparing our initial SLAH subjects to a near-consecutive cohort of open resection patients on a standard verbal memory measure.

## Methods and Materials

### Subjects

We present pre- and postsurgical data for the first 40 patients undergoing SLAH at Emory University, and 40 patients undergoing traditional open TL resections at either Emory University or the University of Washington. Patients were at least 16 years of age and native English speakers. MRI analysis was performed by three subspecialty-certified neuroradiologists experienced in epilepsy imaging. On baseline MRI exams, high-resolution coronal oblique T2-weighted and T2-FLAIR images were used to evaluate signal intensity, volume, and architecture of mesial TL structures. MTS was defined by the presence of hippocampal atrophy plus either abnormal high signal intensity on T2 and/or T2-FLAIR imaging, or blurring of internal architecture (13). Language dominance was determined using fMRI of language and the intracarotid amobarbital (Wada) procedure in cases of elevated memory risk (14, 15). All but eight SLAH patients and three open resection patients were left hemisphere dominant for language. These atypical language patients were grouped by side of language dominance for assessment of change following surgery, and these analyses were completed without their inclusion to ensure that their presence was not unduly affecting outcome. Additionally, side of surgery and language laterality were also explored using multivariable statistical analysis at the group level. Seven additional patients undergoing SLAH at Emory University were excluded from this study based on having undergone a prior open resection surgery (*n* = 3), an unwillingness to participate in our study (*n* = 2), invalid post-surgical neuropsychological testing (*n*=1), or an inability to complete cognitive assessment before undergoing a subsequent open resection (*n* = 1). Details of the SLAH approach are presented in a published technical report (16) and 1-year seizure outcome data are provided in a publication specifically examining this topic in a cohort of SLAH patients that included those in this manuscript (17).

All patients completed pre-surgical neuropsychological evaluation and a 3 Tesla brain MRI. Seizure onset was determined using long-term video-EEG monitoring, and additional invasive electrode monitoring in 9 of 40 patients undergoing open resection and 12 of 40 undergoing SLAH. The latter patients underwent bilateral placement of strip and depth electrode arrays (*n* = 7) or *foramen ovale* electrodes (*n* = 5) that implicated the medial TL as the only seizure onset zone. The nine open resection patients who underwent invasive monitoring had placement of subdural grid electrode arrays with additional strip electrodes. All Emory University patients underwent positron emission tomography (PET) and many University of Washington patients underwent ictal single-photon emission computed tomography (SPECT). All patients were assessed ~1 year following surgery.

For both the Emory and UW sites, inclusion criteria for this study included unilateral seizure onset as determined by the aforementioned EEG studies, surgical treatment with either SLAH or open resection, and available pre- and post-surgical data. As noted, surgical decisions were made on the basis of a review of EEG data (at times including intracranial monitoring), neuroimaging data (structural and functional techniques), and neuropsychological testing results.

Once the SLAH procedure became available at Emory University, patients with focal unilateral medial TL seizure onset were offered a choice of open resection or SLAH, without exception. Therefore, while this was not a randomized trial, patients were able to choose one procedure vs. the other. However, most patients chose SLAH after being presented with both options due to the less invasive nature of the procedure.

Patients were classified by surgery type (open resection vs. SLAH) and laterality of the procedure (dominant vs. non-dominant). Groups did not differ significantly in age of seizure onset, age at surgery, MTS status, or number of prescribed anti-seizure medications (ASMs) (Table 1). Educational attainment was higher for the non-dominant open resection group compared to the dominant open resection group, but this would not affect the planned analyses. ASM regimens did not differ significantly across assessments, as most patients are maintained on their pre-surgical regimen for 1–2 years post-surgically at our epilepsy centers. Seizure free rates also did not differ between surgical cohorts, as can be seen from Table 2.

**Table 1:** Demographic, disease-related variables, surgical characteristics, and test performances by surgical group.

	**Standard open resections**		**Stereotactic laser amygdalohippocampotomy**		**Significance**
**Side of surgery**	**19 Dominant/21 Non-dominant**		**19 Dominant/21 Non-dominant**		***n.s*.**
	**Dominant**	**Non-dominant**	**Dominant**	**Non-dominant**	
	***X SD* (Range)**	***X SD* (Range)**	***X SD* (Range)**	***X SD* (Range)**	
Age (years)	36.9 12.4 (21–59)	39.2 10.8 (22–58)	39.3 17.0 (16–67)	40.7 14.3 (21–64)	*n.s*.
Education (years)	12.4^i^ 2.2 (9–18)	15.1^i^ 2.4 (9–19)	13.4 3.2 (8–20 years)	13.9 2.3 (10–18)	*F*_(3, 76)_ = 3.9, *p < 0.0*2
Age of onset	21.2 14.2	18.8 12.3	14.6 10.1	18.7 14.9	*n.s*.
MTS	10/19	10/21	14/19	10/21	*n.s*.
RAVLT−5-trial total (Pre)	39.1 11.8	43.8 7.8	38.0 10.6	43.9 9.8	*n.s*.
RAVLT−5 trial total (Post)	30.1^ακδ^ 7.1	45.9^αβ^ 9.2	36.7^κβγ^ 10.4	46.5^δγ^ 8.1	*F*_(3, 76)_ = 15.1, *p < 0.0*01
RAVLT–delay (Pre)	6.2 4.2	7.2 4.0	4.8 3.7	6.3 4.0	*n.s*.
RAVLT–Delay (Post)	2.6^Σν^ 2.3	7.3^Σκ^ 4.9	4.4^κ*o*^ 3.3	*8.4^ν*o*^* 3.7	*F*_(3, 76)_ = 10.1, *p < 0.0*01

*Standard resections included both standard and tailored anterior temporal lobe resections and selective amygdalohippocampectomies. Statistical analysis included Fisher exact test comparisons for categorical variables and analysis of variance for continuous data. Matching superscripts indicate that differences between subgroups are significant. Test performance is listed in z scores. MTS, mesial temporal sclerosis; RAVLT, Rey Auditory Verbal Learning Test; Pre, presurgical testing; Post, post-surgical testing*.

**Table 2:** Change in verbal memory by surgery type, laterality of procedure, and seizure outcome using reliable change and standard deviation methodologies.

**Dominant TL procedures**	**Verbal memory outcome–same or improved**	**Verbal memory outcome–declined**
	**RCI vs. (1 SD)**	**RCI vs. (1 SD)**
Engel I	SLAH=9 (9); Open=3 (1)	SLAH=1 (1); Open=6 (8)
Engel II	SLAH=3 (2); Open=2 (1)	SLAH=0 (1); Open=1 (2)
Engel III	SLAH=4 (3); Open=3 (0)	SLAH=1 (2); Open=1 (4)
Engel IV	SLAH=1 (1); Open=2 (2)	SLAH=0 (0); Open=1 (1)
Summative data—without regard to seizures status	SLAH=17/19 Open=10/19(15/19) (4/19)	SLAH=2/19 Open=9/19(15/19) (4/19)
**Non-dominant TL procedures**	**Verbal memory outcome—same or improved**	**Verbal memory outcome—declined**
	**RCI vs. (1 SD)**	**RCI vs. (1 SD)**
Engel I	SLAH=8 (8); Open=11(8)	SLAH=0 (0); Open=1 (4)
Engel II	SLAH=5 (5); Open=2 (2)	SLAH=0 (0); Open=1 (1)
Engel III	SLAH=4 (3); Open=0 (0)	SLAH=0 (1); Open=1 (1)
Engel IV	SLAH=4 (3); Open=5 (4)	SLAH=0 (1); Open=0 (1)
Summative data—without regard to seizures status	SLAH=21/21 Open=18/21 (19/21) (14/21)	SLAH=0/21 Open=3/21 (2/21) (7/21)

*TL, temporal lobe; SLAH, selective laser amygdalohippocampotomy. Significant change was based on both reliable change scores (RCIs) and standard deviation methodology (1 SD), with the latter presented in parenthesis. Verbal memory outcome was significantly better for dominant SLAH patients as compared to dominant open resection patients using either method (p < 0.05, Fisher's exact test). See Methods for a description of the Engel's seizure classification system (Class I outcome is best, representing a seizure free status after surgery)*.

This research project was reviewed and approved by the institutional review boards of Emory University School of Medicine and the University of Washington School of Medicine. All patients provided their written informed consent to participate in this study.

### Surgical Parameters

All MRI-guided SLAH procedures were performed at Emory University. This consists of stereotactic trans-occipital to mesial temporal insertion of a saline-cooled cannula with fiber optic under general anesthesia, targeting the inferior amygdala, the hippocampus from the head to posterior body (mean hippocampal ablation length was 2.5 cm), and the associated uncus (16). Laser-induced interstitial thermal energy was delivered during continuous MRI-based thermography (Visualase, Medtronic, Inc., Louisville, CO). Ablation extent was determined in real-time from thermal imaging and confirmed post procedure with contrasted anatomic imaging.

Open resections consisted of an anterior temporal lobectomy including medial temporal resection that was classified as either standard (3.5 cm lateral temporal resection, *n* = 1) or “tailored” (typically less extensive and guided by intraoperative interictal epileptiform activity, with limited superior temporal gyrus resection, *n* = 26), or transcortical “selective” amygdalohippocampectomy (via inferior temporal sulcus) and parahippocampectomy (*n* = 13). Patients at the University of Washington usually underwent a tailored temporal lobectomy (*n* = 26: 13 dominant/13 non-dominant), with electrocorticography and speech mapping for language dominant patients (18, 19) to determine extent of lateral (superior, middle, inferior), basal temporal cortex, and hippocampal resection. Superior temporal gyrus resection was avoided except for the anterior 1 cm included in the temporal pole. When minimal lateral cortex was involved in the pathology, or in cases done without electrocorticography, only the anterior 3–4 cm of middle and inferior temporal cortex was resected to allow entry into the temporal horn of the lateral ventricle. The inferior lateral amygdala was resected to the roof of the ventricle and the uncinate gyrus was resected in a subpial fashion. On average, the extent of lateral resection in the tailored procedures, was 4.09 cm (*SD* = 1.08; range = 2.4–6.5 cm), with the larger resections tending to occur in the non-dominant hemisphere. Basal TL was resected including parahippocampus and the hippocampal/parahippocampal resection was taken posteriorly to the level of the tectal plate, or less aggressively if indicated due to focal pathology or electrocorticography.

Thirteen patients (6 dominant/7 non-dominant) underwent open “selective” amygdalohippocampectomy and parahippocampectomy at Emory University. This procedure involved exposure to the temporal horn of the lateral ventricle through the inferior temporal sulcus to avoid resecting lateral TL structures. The medial resection was essentially the same as described above, except it always extended to the level of the tectal plate. Parahippocampal gyrus was also resected, but usually not as far posteriorly as was the hippocampus, and the fusiform gyrus was variably included.

**Figure 1:**
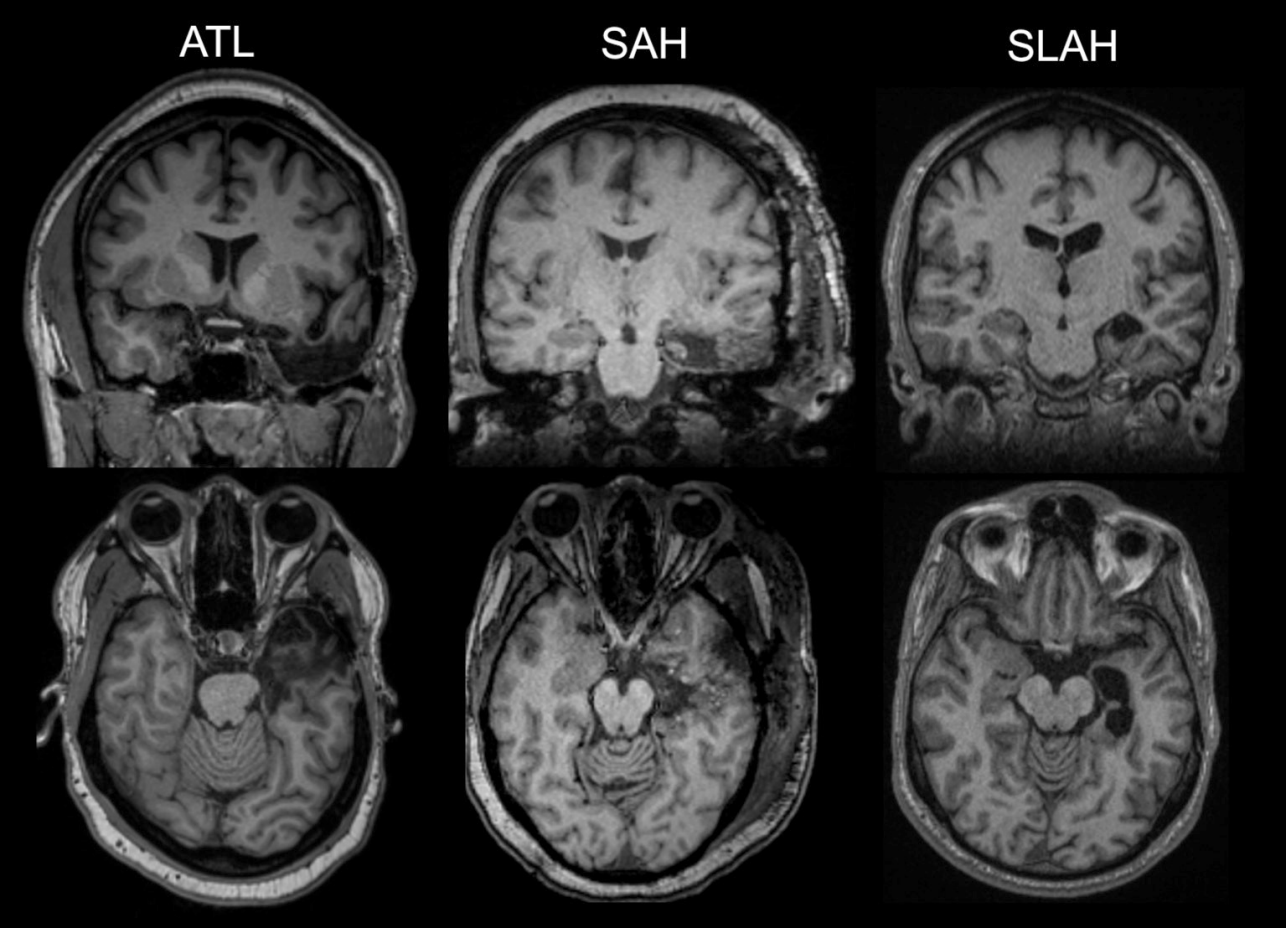
Comparison of selective laser ablation of amygdalohippocampal complex and open resections of the anterior temporal lobe. The first MRI image in each row represents an anterior temporal lobectomy (ATL). The second MRI image is a transcortical selective amygdalohippocampectomy and parahippocampectomy (SAH). The third images on the far right depict a stereotactic laser amygdalohippocampotomy (SLAH), which completely spares the lateral and anterior temporal structures. The MRI images in the top row are from a coronal viewpoint and those in the bottom review are an axial representation. All images are presented using standard neuroradiological convention. (left side of image represents right hemisphere of brain and vice versa). All represent left TL procedures.

Thus, the alternative open temporal resections variously affected several TL regions, but all had in common resection of the amygdalohippocampal complex with uncinate/entorhinal/parahippocampal cortices removed to a point posteriorly between the landmarks of the lateral mesencephalic sulcus and tectal plate, and transection of the temporal stem at some level (Figure 1). More extensive resections also included outright resection of the anterior temporal stem variably affecting white matter paths (e.g., uncinate fasciculus, inferior longitudinal fasciculus, inferior fronto-occipital fasciculus). Tailored and standard lobectomies also variably resected or transected temporal pole, fusiform gyrus and lateral temporal gyri. By comparison, SLAH directly damaged only the amygdalohippocampal complex (amygdala, hippocampus, uncinate gyrus, and subiculum).

### Memory Measures

The Rey Auditory Verbal Learning Test (RAVLT) (20) was administered to all patients. It requires free recall of words from a list of 15 unrelated words, repeated over five separate trials. Recall is tested after each of 5 trial presentations. An alternative word list is then presented and tested, followed by an immediate recall of the words from the original list. This is followed by another free recall at the end of a 30-min delay period filled with other unrelated tasks.

### Statistical Analysis

We began by comparing open resection and SLAH at the level of the individual, as these results would be valuable to predict risk of decline for the potential surgical patient. Baseline and 1-year post-operative performances were compared for learning and delayed recall subscores from the RAVLT. Reliable change index (RCI) scores were used to determine significant change, given their common use in epilepsy research (21, 22). However, RCI scores can obscure meaningful change due to a variety of issues (23, 24). For example, baseline scores are often too high or too low to achieve these RCI score thresholds, making “significant” change impossible in some patients. Moreover, as evidence emerges that interictal epileptiform discharges (IEDs) can affect cognitive performance in a transient yet substantial manner, test-retest indices in epilepsy patients without consideration of IEDs during testing are potentially confounded (25–28). Therefore, we conducted a secondary analysis of outcome using a 1 SD threshold of change based on clinical normative data. This latter approach has been used in several of the existing SLAH studies (5, 6, 29), as well as many epilepsy outcome studies with open resection and other therapeutic devices (30–32).

Fisher's exact test was used to compare cognitive change on the RAVLT subscores in the surgical groups. These analyses were completed without regard to side of surgery, to examine the rate of significant decline on one or both measures in the entire sample. These analyses were repeated with grouping by each of the four different combinations of surgery type (SLAH and open resection) and laterality (dominant and non-dominant). Baseline and post-surgical memory measures were also compared after separating patients by side and type of surgery, using an analysis of variance (ANOVA) test. There were significant pre-operative group differences on verbal learning and memory measures (Table 1), favoring the non-dominant over the dominant surgery patients. There were no significant pre-surgical differences on memory measures between groups based upon surgical technique (i.e., ablation vs. open resection).

At the group level, we used paired-sample *t*-tests to determine if significant change occurred from pre- to post-surgical evaluation on any measure for each subgroup. Additionally, as a secondary analysis to potentially inform future research, we explored possible predictors of group level change using multiple regression analysis, as we suspected that different combinations of factors would contribute to whether a patient improved or declined on these memory measures following surgery. Predictors of interest included age of seizure onset, age at the time of testing, presence or absence of MTS on MRI, side of surgery, type of surgery (SLAH vs. open resection), language laterality, and seizure freedom status. Several studies have suggested that one needs at least 10 subjects per variable to avoid model over-fitting, and thus our sample size appears adequately powered for this analysis (33, 34).

## Results

### Individual Level

**Figure 2:**
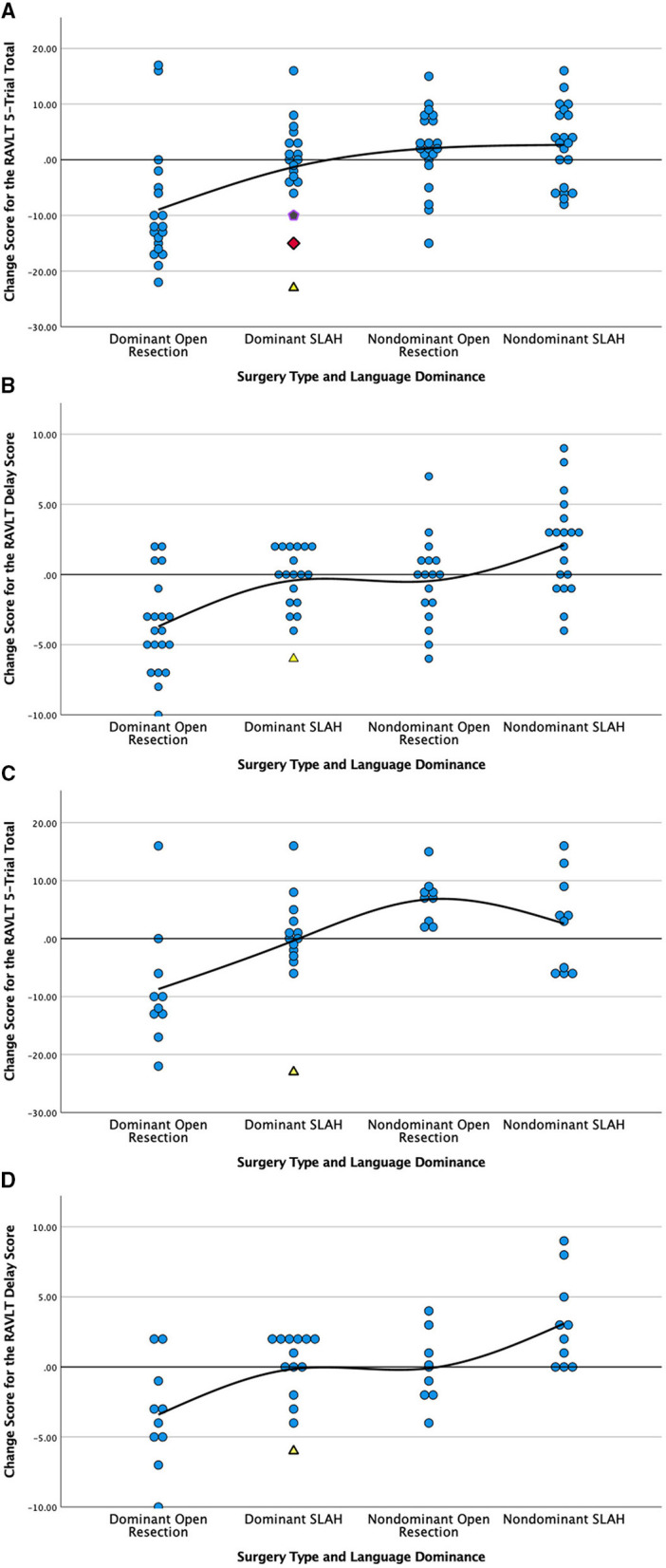
Comparison of post-surgical change in verbal memory by surgery type and language dominance for all patients **(A,B)** and only those with MTS **(C,D)**. Change is presented in raw scores as scatter plots grouped by type of surgery and laterality of seizures. Verbal Learning, RAVLT 5-Trial Total Score; Verbal Delay, RAVLT Delayed Recall Score; RAVLT, Rey Auditory Verbal Learning Test; SLAH, stereotactic laser amygdalohippocampotomy; MTS, mesial temporal sclerosis. Open temporal lobe resections involve the open anterior temporal lobectomy and selective amygdalohippocampectomy procedures described in methods. Scores above the solid line represent improvement on the measure and scores below represent decline. **(A)** Change in verbal learning score by type and side (laterality) of surgery for all patients; **(B)** Change in delayed verbal recall by type and side (laterality) of surgery for all patients; **(C)** Change in verbal learning score by type and side (laterality) of surgery for MTS patients only; **(D)** Change in delayed verbal recall by type and side (laterality) of surgery for MTS patients only. We have used alternative symbols for those SLAH patients showing significant decline on memory measures in order to highlight that each had either wider-spread structural brain abnormalities (i.e., yellow triangle represents a patient with dual pathology involving the left lateral TL and the purple pentagram represents a patient with bilateral structural abnormalities) or normal neuroimaging (red diamond) in a single case.

Using RCI scores, open temporal resection in either cerebral hemisphere was more likely to cause a significant decline on a measure of verbal memory (RAVLT) than SLAH (12/40 [30.0%] vs. 2/40 [5.0%], *p* = 0.0064, Fisher's exact test). This distinction was driven by open resections in the language dominant (typically left) temporal lobe (dominant open resections: 9/19 [47.4%] vs. dominant SLAH: 2/19 [10.5%], *p* = 0.0293, Fisher's exact test) (Table 2 and Figure 2).

When using the 1 SD method of determining significant change, the same pattern of greater verbal memory decline following open TL resection vs. SLAH was observed. However, as this metric of change is less stringent, more patients exhibited decline in both groups. Once again, using the 1 SD metric, open resection involving either cerebral hemisphere was more likely to cause a significant verbal memory decline than SLAH (22/40 [55.0%] vs. 6/40 [15.0%], *p* = 0.0003, Fisher's exact test). This distinction between procedures was again greater for open resection in the language dominant TL (dominant open resections: 15/19 [78.9%] vs. dominant SLAH: 4/19 [21.1%], *p* = 0.0009, Fisher's exact test) (Figure 2 and Table 2). Strikingly, 1 SD verbal memory decline frequently occurred in the open resection sample of dominant TL patients with MTS (8/10 [80.0%]), although it rarely occurred in such patients after SLAH (2/14, 14.3%) (*p* = 0.0027, Fisher's exact test; Figure 2).

Post-surgical improvement in verbal memory with RCI methodology was rarely observed in either group. This included 4/40 patients following SLAH (1 dominant/3 non-dominant) and 3/40 patients following open resection (2 dominant/1 non-dominant). In contrast, several SLAH patients showed at least a 1 SD gain in verbal memory (15/40: 3/19 dominant and 12/21 non-dominant), with this improvement observed significantly more often in this patient group as compared to the open resection sample (6/40: 2/19 dominant and 4/21 non-dominant; *p* = 0.0406, Fisher's exact test). Overall, verbal memory functioning of non-dominant TL patients was more likely to improve following SLAH than open resection.

Of the two SLAH patients who declined on RCI analysis, one patient exhibited no evidence of MTS or any other pathology (“normal” MRI), and one had preoperative imaging evidence of MTS plus additional pathology in the lateral TL region (e.g., temporal pole dysplasia and loss of gray-white matter differentiation). Both patients were in the language dominant group. The 1 SD analysis identified these same two patients as declining, as well as two additional language dominant patients and two non-dominant patients. The two additional language dominant cases that declined included one patient with left MTS only and one with bilateral hippocampal abnormalities (i.e., small left hippocampus and signal change involving the right hippocampus) and broader pathology, including right frontal periventricular encephalomalacia thought to reflect a remote ischemic event, and a right parietal arachnoid cyst. The two non-dominant SLAH cases declining more than 1 SD included one patient with normal neuroimaging and one with bilateral inflammatory changes involving the temporo-parietal regions and a normal hippocampus. Although the latter patient no longer exhibited seizures arising from his non-dominant hemisphere following right SLAH, he began experiencing seizures from his dominant hemisphere. Therefore, it is possible that post-ablation decline was related to a new seizure focus in the unoperated cerebral hemisphere.

Table 3 provides a breakdown of whether individual patients exhibited significant improvement or decline on any subsection of the RAVLT examined in this study. Outcome was presented with both RCI and the 1 SD metric of change. The open resection patients were more likely to decline on both immediate and delayed recall measures regardless of metric of change. When decline occurred in SLAH patients, it tended to be on delayed recall only.

**Table 3:** Patterns of verbal memory decline observed in open resection and SLAH based on both reliable change scores and single standard deviation methodologies.

**Type of surgery**	**Left TL open resection**	**Left SLAH**	**Right TL open resection**	**Right SLAH**
	**(*n* = 19)**	**(*n* = 19)**	**(*n* = 21)**	**(*n* = 21)**
Decline on 5-trial learning only	1 (0)	1 (1)	0 (0)	0 (0)
Decline on delayed recall only	3 (1)	0 (1)	1 (4)	0 (2)
Decline on both verbal memory measures	5 (13)	1 (2)	1 (2)	0 (0)
No decline on either verbal memory measure	10 (5)	17 (14)	19 (14)	21 (19)
Improvement on 5-trial learning and decline on delayed recall	0 (1)	0 (1)	0 (0)	0 (0)
Decline on 5-trial learning and improvement on delayed recall	0 (0)	0 (0)	0 (1)	0 (0)
Improvement on 5-trial learning only	2 (2)	1 (2)	0 (1)	0 (2)
Improvement on delayed recall only	0 (0)	0 (0)	1 (1)	2 (6)
Improvement on both verbal memory measures	0 (0)	0 (1)	0 (2)	1 (4)
No improvement on either verbal memory measure	17 (17)	18 (16)	20 (17)	18 (9)

*SLAH, selective laser amygdalohippocampotomy; TL, temporal lobe. Significant decline was based on both reliable change index scores (RCIs) and standard deviation methodology (1 SD), with the latter presented in parenthesis*.

### Group Level

At the group level, based on the results of paired sample *t* tests, dominant open TL resection patients declined significantly on both immediate [*t*_(18)_ = 3.69, *p* = 0.002] and delayed verbal list learning scores [*t*_(18)_ = 4.56, *p* < 0.001] following surgery, while the dominant SLAH patients did not (Table 1). Non-dominant SLAH patients exhibited a significant improvement at the group level on the delayed verbal recall measure [*t*_(20)_ = −2.82, *p* = 0.011]. No other groups exhibited significant improvement on either verbal memory measure at the group level.

Multiple regression analyses were completed to determine which factors contributed to significant change in performance on the 5-trial learning and delayed recall measures of the RAVLT. For both of these measures, performance change was predicted by baseline performance on the task (*p* < 0.001), side of surgery (*p* < 0.001), and type of surgery (*p* = 0.001). In contrast, age of seizure onset, age at time of surgery, Engel's outcome, and cerebral language dominance did not predict change in performance for either 5-trial learning or recall. The overall models were significant for both 5-trial learning, [*F*_(7, 72)_ = 7.56, *p* < 0.001, *R*^2^ = 0.424], and for delayed recall, [*F*_(7, 72)_ = 8.43, *p* < 0.001, *R*^2^ = 0.450], of the RAVLT.

All proportional change by surgical procedure analyses were repeated with the 11 patients with atypical language lateralization removed to ensure that outcome was not affected by these individuals. There were no changes in the pattern of findings, with all results unchanged when considering the left language dominant patients only (*n* = 69). In fact, one of the six SLAH patients to decline at the 1 SD level had atypical language, so the results were actually strengthened (see Table 4). Similarly, as another check on the potential influence of seizure outcome, we examined rates of decline for patients with only Engel's 1a outcome. For these patients, 7 of 8 OR patients experienced significant decline on one or both RAVLT outcome measures while only 1 of 7 SLAH patients exhibited decline.

**Table 4:** Change in verbal memory by surgery type, laterality of procedure, and seizure outcome excluding atypical language cases.

**Dominant TL procedures**	**Verbal memory outcome—same or improved**	**Verbal memory outcome—declined**
	**RCI vs. (1 SD)**	**RCI vs. (1 SD)**
Engel I	SLAH=7 (7); Open=2 (1)	SLAH=1 (1); Open=6 (7)
Engel II	SLAH=2 (1); Open=2 (0)	SLAH=0 (1); Open=1 (2)
Engel III	SLAH=2 (1); Open=1 (0)	SLAH=1 (2); Open=1 (1)
Engel IV	SLAH=0 (0); Open=1 (2)	SLAH=0 (0); Open=0 (1)
Summative data—without regard to seizures status	SLAH=11/13; Open=6/14 (9/13) (3/14)	SLAH=2/13; Open=8/14 (4/13) (11/14)
**Non-dominant TL procedures**	**Verbal memory outcome—same or improved**	**Verbal memory outcome—declined**
	**RCI vs. (1 SD)**	**RCI vs. (1 SD)**
Engel I	SLAH=5 (5); Open=9 (5)	SLAH=0 (0); Open=1 (0)
Engel II	SLAH=2 (2); Open=1 (1)	SLAH=1 (1); Open=0 (1)
Engel III	SLAH=2 (2); Open=0 (0)	SLAH=0 (0); Open=0 (1)
Engel IV	SLAH=1 (1); Open=2 (4)	SLAH=0 (0); Open=0 (1)
Summative Data—without regard to seizures status	SLAH=10/11; Open=10/13 (10/11) (12/13)	SLAH=1/11; Open=1/13 (1/11) (3/13)

*TL, temporal lobe; SLAH, selective laser amygdalohippocampotomy. Significant change was based on both reliable change index scores (RCIs) and standard deviation methodology (1 SD), with the latter presented in parenthesis for the cumulative totals only. While the magnitude of change is smaller with the standard deviation methodology. Verbal memory outcome was significantly better for dominant SLAH patients as compared to dominant open resection patients using either method even after atypical language patients were removed from consideration (p < 0.05, Fisher's exact test). See Methods for a description of the Engel's seizure classification system (Class I outcome is best, representing a seizure free status after surgery)*.

## Discussion

Epilepsy surgery patients undergoing highly selective stereotactic laser amygdalohippocampotomy experience a better outcome on a standard verbal list learning task as compared to a comparable cohort of open resection patients. SLAH patients are less likely to decline on this verbal memory measure, and non-dominant SLAH patients are more likely to improve on this task following surgery than are open resection counterparts. Language dominant TL open resection patients exhibited a significant decline at the group level for both learning and recall measures of this standard verbal list learning task, with their final group mean falling significantly below the SLAH cohort. While decline in verbal memory can occur following SLAH, it is less severe in magnitude and occurs less frequently than in open resection patients. These findings were observed in the entire sample, and also remained strong when considering only patients who experienced the most optimal seizure outcome (i.e., Engel 1a).

Some prior studies of SLAH outcome have reported verbal memory decline (6, 29, 35), but they have typically lacked a control group or a review of the historical literature on open resections. We have demonstrated in a review of the literature that aggregating the frequency of decline across the initial SLAH outcome reports showed that verbal memory was declining in ~15% of patients (7), clearly below historical estimates of 30-60% rates of decline for open resection (36, 37). Additionally, several of these reports of memory outcome following SLAH used a 1 SD metric of change, which is more likely to find change than outcome studies using RCI or other statistical methodologies for determining significance. Our current data support this same pattern of outcome, and highlights how the use of differing statistical methods of describing change can produce highly variable range of decline. The use of RCI metrics resulted in a more conservative estimate of verbal memory decline for both procedures (i.e., 30% of open resection patients and 5% of SLAH patients) than did the 1 SD method (i.e., 55% of open resection patients and 15% of SLAH patients). While many have argued for use of RCI measures, we believe that even smaller metrics of change can be relevant and have a meaningful effect on the lives of patients. Additionally, as we have argued elsewhere, RCI metrics are potentially confounded by the likely presence of interictal epileptiform activity during their development, as there was no simultaneous EEG obtained during their creation (25, 28). Additionally, making a comparison to other patients with intractable epilepsy may underestimate the recovery capacity of patients who become seizure free (23, 24). Many of our SLAH patients show improvements on some tasks more in line with healthy controls rather that of epilepsy patients who remain intractable (7).

Since SLAH and open resection groups did not significantly differ on clinical factors (age, ASMs, type, severity and duration of seizures), the observed between-group difference appears to result from the sparing by SLAH of extrahippocampal structures and pathways disrupted during open resection. These include the temporal pole, lateral temporal gyri, perirhinal and entorhinal cortices, parahippocampal gyrus, fusiform gyrus, and temporal white matter tracts. Even selective amygdalohippocampectomy still entails retraction or partial transection of lateral temporal gyri, transection of white matter pathways in the temporal stem, and resection of the parahippocampal region (38), structures spared with the laser approach. Overall, it is likely that achieving seizure control with less collateral injury better preserves cognitive function and aids functional recovery.

Our findings confirm numerous animal studies and human experimental paradigms, which suggest the amygdalohippocampal complex is only a part of a broader medial TL network involved in memory formation (12, 39–42), extending this concept to a clinical sample of epilepsy surgery patients. More specifically, the cognitive impact of injury to the structures connected to and surrounding the hippocampus, such as the parahippocampal gyrus and perirhinal area, better model human amnesia, than injury to the hippocampus and amygdala alone (10, 12). The structures typically deemed important for the support of declarative memory have been the hippocampus and the entorhinal, perirhinal, and parahippocampal cortices. Based on non-human primate neuroanatomical tracing, the hippocampal formation has mostly direct reciprocal connections with these areas, and Squire and others have suggested that the hippocampus may integrate these inputs (e.g., binding stimulus properties to objects or objects to context) (41).

In addition to non-human primate and clinical findings with medial temporal injury, lateral temporal lesions can also impair memory. For example, Ojemann et al. used a stimulation mapping procedure to demonstrate that verbal memory could by impaired by stimulating aspects of the fusiform gyrus (43). Additionally, the extent of resection of lateral, not medial, temporal lobe structures correlates with degree of verbal memory decline (44). Our SLAH results represent an extreme variant of this observation, with no lateral, fusiform or parahippocampal structures ablated, supporting the argument that post-surgical verbal memory decline is in large part due to additional involvement of extrahippocampal structures that can include subjacent cortex, lateral temporal cortex and temporal white matter.

This argument is also supported by our finding that open TL resection surgery carried a greater risk of postoperative verbal memory decline than SLAH in patients *with or without MTS*. Since both groups of MTS patients have baseline hippocampal dysfunction, greater declines from open surgery implicate extra-hippocampal structures in verbal memory function. This is consistent with observations that patients undergoing limited resection of focal lesions in the fusiform gyrus (e.g., cavernous malformations, focal cortical dysplasias) (40, 45), rather than the hippocampus or other medial temporal structures, often experience significant memory declines, which we have observed with selective stereotactic laser ablation of this region as well (7, 46). In such cases, it is implausible that verbal memory would reorganize from a normal appearing medial temporal region to the fusiform gyrus harboring pathology. The use of minimally invasive ablative procedures in humans coupled with neuroimaging connectivity studies and work in non-human primate or rodent models on memory circuits holds great promise for dissecting the functional anatomy of memory and other cognitive functions.

An exception to the more favorable verbal memory outcomes after SLAH compared to open resection was our finding that a few SLAH patients (i.e., 6 of 40 using the 1 SD metric) with verbal memory decline typically had additional pathology (*n* = 3) in other ipsilateral or contralateral temporal regions (e.g., dysplasias involving the left temporal pole or fusiform gyrus in dominant SLAH cases, bilateral hippocampal abnormalities) or otherwise had normal neuroimaging (*n* = 2). In the first case, it is possible that ablation of medial structures, combined with preexisting dysfunction of broader networks, including lateral temporal cortex, caused the decline in the patients with broader pathology because it resulted in network deficits comparable to the territories affected by traditional open surgeries. However, as for the two SLAH patients with a normal preoperative MRI without MTS who experienced verbal memory decline, the results may implicate the hippocampus in verbal memory, although we believe this role is best seen as part of a much broader neural substrate supporting this function. We also had a third normal neuroimaging patient who declined significantly at 6-month follow-up, but recovered to baseline by 1-year examination. Exploring memory patterns and underlying neural circuitry in these patients over time with functional MRI and various measures of connectivity (e.g., resting state fMRI, DTI), will likely be helpful in determining to what extent postoperative reorganization of function occurs.

Similar to our findings of limited verbal memory decline in cases with normal imaging, there is a single case study of two PET positive, MRI-negative patients that also reported possible declines in memory (5). These patients were assessed during acute stage recovery without subsequent psychometric follow-up, although the researchers report what sounds like a good functional recovery over time. It is possible that further improvement may have occurred in these patients, as we observed in one of our normal imaging SLAH patients. Although our findings indicate that verbal memory is sustained by broad dominant temporal neural networks, and that the hippocampus is more dispensable to verbal memory function than generally accepted in clinical practice, we advise caution when considering any surgical procedure involving the language dominant, amygdalohippocampal complex in an MRI normal individual with normal baseline memory. As we learn more about the specific neural circuits of memory and their capacity for reorganization following minimal surgical perturbations, it should be possible to more knowledgeably counsel patients to the potential risks in such cases. It may eventually be possible to consider minimally invasive interventions in more than one location or even both cerebral hemispheres once neural circuitry supporting major cognitive and behavioral functions is better understood.

It will also be important to study variability in the use of the SLAH procedure (extent and location of ablations), as some neurosurgery groups may choose to ablate more broadly (i.e., include other medial TL structures) and variability in these parameters occur even across the cases of a single neurosurgeon. It may be possible to determine optimal placement of the optic fiber to optimize seizure control while avoiding cognitive side effects or other adverse sequelae (e.g., visual field cuts, cranial nerve injury)(47, 48). The use of structural volumetry, DTI tractography, and resting state fMRI coupled should be explored in relation to outcome metrics in future studies.

The emerging view that episodic memory is more heterogeneous than previously understood also deserves discussion (40). Although we were less likely to detect significant verbal memory declines from selective amygdalohippocampotomy alone, this may reflect a limitation of currently available verbal memory tasks, highlighting a potential need for improved tasks better optimized to discriminate potential hippocampal and non-hippocampal functions. Such tasks might include multimodal binding of linguistic, semantic, and sensory information or perhaps relating temporal and contextual features of verbal information in a learning paradigm. Likewise, tasks that create more demand for spatial recall may be more affected by isolated hippocampal damage given historical research in the setting of non-human primates and rodents demonstrating a significant role for this structure in spatial processing and route learning (49, 50).

Although the open resection and SLAH cohorts did not significantly differ on the basis of age at time of surgery, the SLAH group contained two patients in their mid- to upper 60s, while there were no patients in this age range in the open resection group. Both patients indicated that they had been offered open resections previously, but had declined. This was reported by several SLAH patients, and reflects that more patients may be willing to consider this procedure for treatment of their epilepsy.

Limitations of the current study include a non-randomized trial, a relatively small sample size, and only two epilepsy centers where the distribution of SLAH is uneven. In contrast, this does represent the first direct comparison of SLAH and open resection procedures for memory. Moreover, we employed a commonly used, well-standardized verbal memory measure known to be sensitive to TL damage with a well-defined epilepsy surgical cohort from two level 4 epilepsy centers with assessment of several potential contributing or confounding variables.

We previously reported that the highly selective SLAH procedure results in less dysfunction in confrontation naming and object and face recognition (1). The current study extends this work by demonstrating that removal of the amygdalohippocampal complex alone, compared to broader open TL resections, also yields superior outcomes on standard measures of verbal episodic memory, challenging a long-held clinical belief that this function critically depends upon the hippocampus. SLAH resulted in fewer patients with post-surgical declines, and more patients undergoing SLAH (particularly non-dominant) exhibited improvements in verbal memory.

These findings provide further evidence for the importance of extra-hippocampal temporal cortex and white matter in verbal memory. Additional studies with larger sample sizes are needed to confirm these findings on cognitive outcomes and to define the relative efficacy for seizure freedom between SLAH and open resections. This is needed to define the relative risk/benefit ratio of the two procedures for patient subgroups.

## Data Availability Statement

The datasets presented in this article are not readily available because there are some restrictions imposed by our Institutional Review Board on sharing data in an online repository, but in general we will make every effort to provide a de-identified dataset to interested investigators upon request. Requests to access the datasets should be directed to Daniel Drane, ddrane@emory.edu.

## Ethics Statement

The studies involving human participants were reviewed and approved by Emory University IRB. We obtained written informed consent from each participant or their legal representative.

## Author Contributions

DD, JW, NV, JO, DL, KM, and RG contributed to conception and design of the study. DD, JW, NP, MK, KH, JO, and RG contributed to data collection on the project. DQ, NV, AS, and TG contributed to planning of MRI sequences. DD, MK, and KH organized the database. BS, AS, and RH analyzed all neuroimaging data. RH selected all representative MRI images used in Figure 1 and prepared this figure. JW, JO, and RG performed all neurosurgical procedures reflected in this manuscript. DD and SM performed the statistical analysis. DD wrote the first draft of the manuscript, organized, and oversaw the overall project. All authors contributed to manuscript revision, read, and approved the submitted version.

## Funding

This project was supported by grants received by DD from the National Institute of Neurological Disorders and Stroke (NINDS) of the National Institutes of Health (NIH) [K23 NSO49100, K02NS070960, L30 NS080215, R01NS088748] and Medtronic, Inc. [A1225797BFN:1056035], as well as a Shared Instrumentation grant (S10: Grant 1@110ODO16143) to the Emory University CSI Core. NP was supported by a CURE Epilepsy Award, NIH NINDS K08NS105929 and R21NS122011.

## Conflict of Interest

Medtronic, Inc., contributed research funding to Emory University, and develops products related to the research described in the paper. However, Medtronic, Inc., was not involved in the study design, data collection, analysis, interpretation of data, the writing of this article or the decision to submit it for publication. RG and JW serve as consultants to Medtronic, Inc., and receive compensation for these services. The terms of this arrangement have been reviewed and approved by Emory University and Washington University in Saint Louis in accordance with their respective conflict of interest policies. The remaining authors declare that the research was conducted in the absence of any commercial or financial relationships that could be construed as a potential conflict of interest.

## Publisher's Note

All claims expressed in this article are solely those of the authors and do not necessarily represent those of their affiliated organizations, or those of the publisher, the editors and the reviewers. Any product that may be evaluated in this article, or claim that may be made by its manufacturer, is not guaranteed or endorsed by the publisher.
